# A short conserved motif in ALYREF directs cap- and EJC-dependent assembly of export complexes on spliced mRNAs

**DOI:** 10.1093/nar/gkw009

**Published:** 2016-01-14

**Authors:** Agnieszka M. Gromadzka, Anna-Lena Steckelberg, Kusum K. Singh, Kay Hofmann, Niels H. Gehring

**Affiliations:** Institute for Genetics, University of Cologne, D-50674 Cologne, Germany

## Abstract

The export of messenger RNAs (mRNAs) is the final of several nuclear posttranscriptional steps of gene expression. The formation of export-competent mRNPs involves the recruitment of export factors that are assumed to facilitate transport of the mature mRNAs. Using *in vitro* splicing assays, we show that a core set of export factors, including ALYREF, UAP56 and DDX39, readily associate with the spliced RNAs in an EJC (exon junction complex)- and cap-dependent manner. In order to elucidate how ALYREF and other export adaptors mediate mRNA export, we conducted a computational analysis and discovered four short, conserved, linear motifs present in RNA-binding proteins. We show that mutation in one of the new motifs (WxHD) in an unstructured region of ALYREF reduced RNA binding and abolished the interaction with eIF4A3 and CBP80. Additionally, the mutation impaired proper localization to nuclear speckles and export of a spliced reporter mRNA. Our results reveal important details of the orchestrated recruitment of export factors during the formation of export competent mRNPs.

## INTRODUCTION

In metazoan cells, synthesis of mature messenger RNAs (mRNA) requires several steps of processing, which are accompanied by the deposition of proteins onto the mRNA to form messenger ribonucleoprotein complexes (mRNPs). Importantly, the composition of mRNPs influences gene expression both in the nucleus and in the cytoplasm and undergoes constant remodelling (1,2). In eukaryotes transcription and translation, the two major steps of gene expression are spatially separated from each other by the nuclear membrane. Therefore, the assembly, nuclear export and subsequent transport of mature mRNPs are prerequisites for the efficient biosynthesis of proteins.

In mammalian cells, splicing plays an important role in mRNP remodelling and thereby influences later steps of gene expression. Consequently, the deposition of export components occurs mainly during splicing (3–5). This is thought to result at least in part from the splicing-dependent deposition of exon junction complexes (EJC), which interact with some of the export factors (6–8). The mammalian TREX complex, which is composed of UAP56, ALYREF and the mammalian THO complex, plays a pivotal role in coordinating splicing with mRNA export (9). ALYREF (also referred to as Aly, REF; in yeast: Yra1p) and its interaction partner UAP56 (BAT1; in yeast: Sub2) were described as components of the EJC (6). Interestingly, UAP56 is also an essential splicing factor, required for the first ATP-dependent step of splicing and spliceosome interaction with the branch point (10,11). A single UAP56 gene is present in yeast and fruit flies, whereas mammalian cells also express DDX39, an additional UAP56-related helicase (12,13). Furthermore, in mammalian cells only the simultaneous depletion of UAP56 and DDX39 causes a substantial retention of mRNAs in the nucleus (14), suggesting a redundant function of these two proteins (12).

According to current models, ALYREF is deposited onto mRNAs through its interaction partner UAP56 (3,15). The formation of export-competent mRNPs involves the splicing-dependent assembly of the THO/TREX complex, of which UAP56 and ALYREF are integral components (15,16). Moreover, the 5′ cap facilitates the recruitment of export factors to intron-containing and intronless mRNA (16–18). Eventually, the binding of the general export receptor NXF1 (Tap) and its heterodimerisation partner NXT1 (p15) to the mRNP initiates the translocation through the nuclear pore (19–21). Several export factors have been identified in mammalian cells, but their mode of interaction with mRNAs and the determinants for their recruitment to the specific mRNP is not understood entirely.

Up to date, several additional mRNA export adaptors have been identified to modulate the process of mRNA export: CHTOP, CIP29, LUZP4 and UIF. Interestingly, CHTOP, LUZP4 and UIF are found to directly interact with UAP56 via a short conserved sequence referred to as UBM (UAP56 binding motif) (22). CHTOP enhances the ATPase activity of UAP56 (23). LUZP4 was found to complement ALYREF knock down *in vivo* and UIF was reported to influence export through binding with NXF1 (22,24). Finally, CIP29 (yeast homologue: Tho1p) interacts with UAP56 and ALYREF, forming a trimeric complex (25,26). Interestingly, CIP29 does not have an UBM. Moreover, it has been suggested that the family of export factors shares a common motif structure, enabling the interaction with UAP56 and possibly other mRNP components to enter the mRNA export pathway (22,24). Thus, the list of export adaptors is not yet complete, and the systematic approach to investigate these is yet to be found.

Short-linear motifs (SLiMs), also referred to as ELM (eukaryotic linear motifs), are short (3–10 amino acid long), conserved sequences situated in intrinsically disordered regions, lacking secondary and tertiary structures. SLiMs mediate low-affinity interactions between proteins and also between RNA and proteins (e.g. RGG and YGG motifs, RS domains, GY-rich regions, etc.) (27–30). Although little is known about the function of low complexity regions in RNA-binding proteins, short linear motifs might be involved in the dynamic rearrangement of mRNPs composition during mRNA export and the assembly of a variety of export adaptors.

In this study, we investigated the interactions of mammalian export components with different RNA substrates. We find that export components UAP56 and ALYREF are recruited to spliced mRNA in an EJC- and cap-dependent manner. We identified four short, conserved, linear motifs present in proteins involved in mRNP processing and export, providing a systematic view into the family of export adaptors. Finally, we show that short linear motif in ALYREF (WxHD) is required for its *in vitro* binding to spliced RNA and for efficient export of spliced mRNAs *in vivo*. Together, these findings expand our view on how nuclear mRNA processing during splicing orchestrates the recruitment of export factors to the RNA and provide insight in to the role of short linear motifs in this process.

## MATERIALS AND METHODS

### Plasmids and cloning

**Figure 1.**
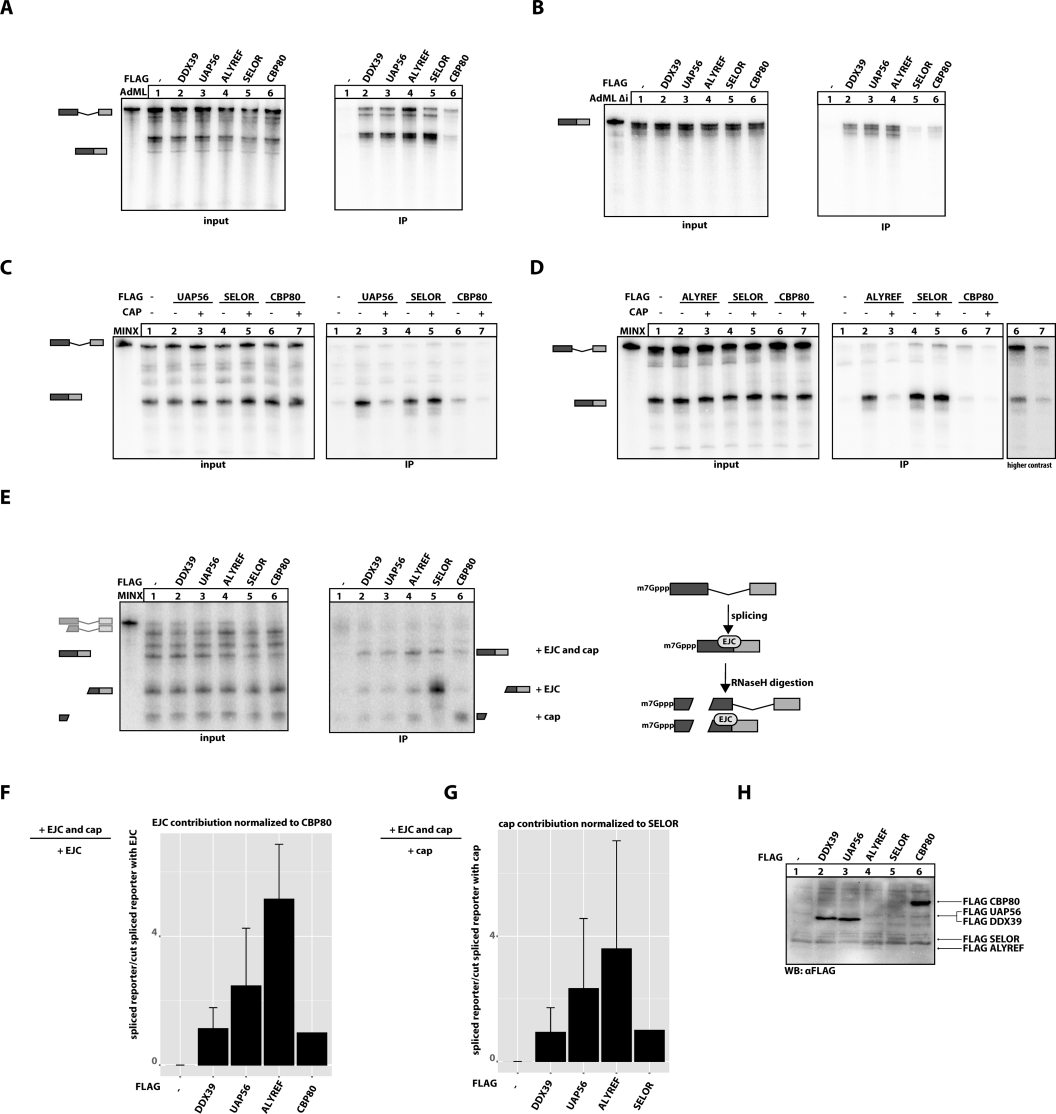
Export factors binding to mRNA during splicing. (**A**) *In vitro* splicing reactions of AdML substrate were supplemented with extracts expressing the indicated FLAG-tagged export factors. SELOR (eIF4A3-binding domain of BTZ) and CBP80 were used as controls, unfused FLAG-tag served as a negative control. FLAG-containing mRNPs were immunoprecipitated with anti-FLAG beads and co-precipitated RNA resolved on a denaturing PAGE. A total of 10% of each splicing reaction was used as input. Schematic representations of splicing products are depicted on the left side of the autoradiograph. (**B**) *In vitro* splicing reactions were performed as in (A) using intronless AdML substrate (AdML Δi). (**C**) *In vitro* splicing reactions were performed as in (A) were supplemented with 10 μM of cap analogue (m^7^GpppG) as indicated and mRNPs were immunoprecipitated and RNAs resolved on denaturing PAGE. FLAG-tagged SELOR and CBP80 served as negative and positive controls, respectively. (**D**) *In vitro* splicing reaction of extract expressing FLAG-tagged ALYREF supplemented with cap analogue was performed as in (C). A high-contrast picture of lanes 6 and 7 is shown as separate panel. (**E**) *In vitro* splicing reactions were performed as in (A) using MINX substrate followed by incomplete RNase H digestion of splicing products and immunoprecipitation of mRNPs. The splicing and digestion scheme is represented on the left side of the autoradiograph. Expression of FLAG-tagged proteins in HEK 293 extracts used in panels A–D was determined by representative immunoblot analysis using a FLAG-antibody. (**F**) Quantification of three independent, biological repetitions of the experiment presented in (E). The EJC contribution to the binding of export components is a calculated ratio of intensities of two bands as depicted on the left side of the graph. EJC contribution to the CBP80 binding serves as a negative control and was set to 1. (**G**) Quantification of three independent, biological repetitions of the experiment presented in (E). The EJC contribution to the binding of export components is a calculated ratio of intensities of two bands as depicted on the left side of the graph. The cap contribution to the SELOR binding serves as a negative control and was set to 1. (**H**) Expression of FLAG-tagged proteins in HEK 293 extracts used in (A–G) was determined by immunoblot analysis using a FLAG-antibody.

**Figure 2.**
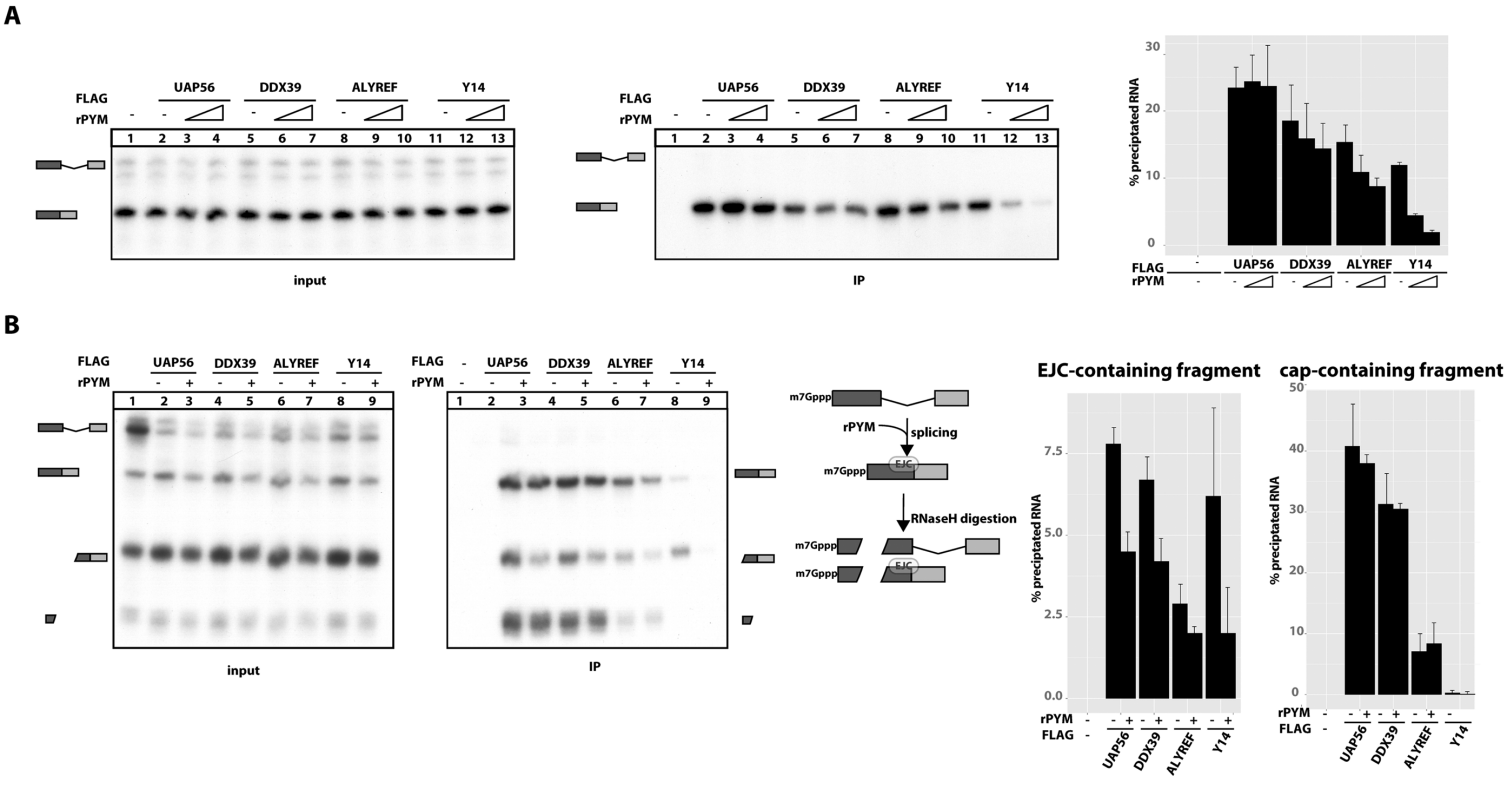
Export factors binding to capped, spliced mRNA is influenced by EJC disassembly by PYM. (**A**) Splicing reactions were performed as described in Figure 1C in the presence of increasing amounts (0, 1 and 2.5 μg) of recombinant PYM (rPYM) as indicated by the triangle. mRNPs were immunoprecipitated using FLAG affinity agarose and analysed by denaturing PAGE. Three independent repetitions of the experiment presented in this panel were quantified and presented on the graph. (**B**) Splicing reactions and oligonucleotide directed RNase H digestions (performed as described in Figure 1E) were carried out in the presence or absence of rPYM as described in Figure 2A. mRNPs were immunoprecipitated using FLAG affinity agarose and analysed by denaturing PAGE. Three independent repetitions of the experiment presented in this panel were quantified and presented on the graphs.

DDX39 and UAP56 were obtained by reverse transcription (RT)-PCR using HeLa cells RNA. ALYREF was amplified by PCR from plasmid DNA (Origene). ALYREF deletion mutants: 1–106, 106-ter, 1–187, RRM, ΔN15, ΔC53 and ΔN15/ΔC53 were generated using PCR. ALYREF 87 WQHD/DQAK 90 was generated by site-directed mutagenesis. Expression vectors for FLAG CBP80, FLAG Y14 and FLAG SELOR were described previously (31) (Figures 1 and 2).

**Figure 3.**
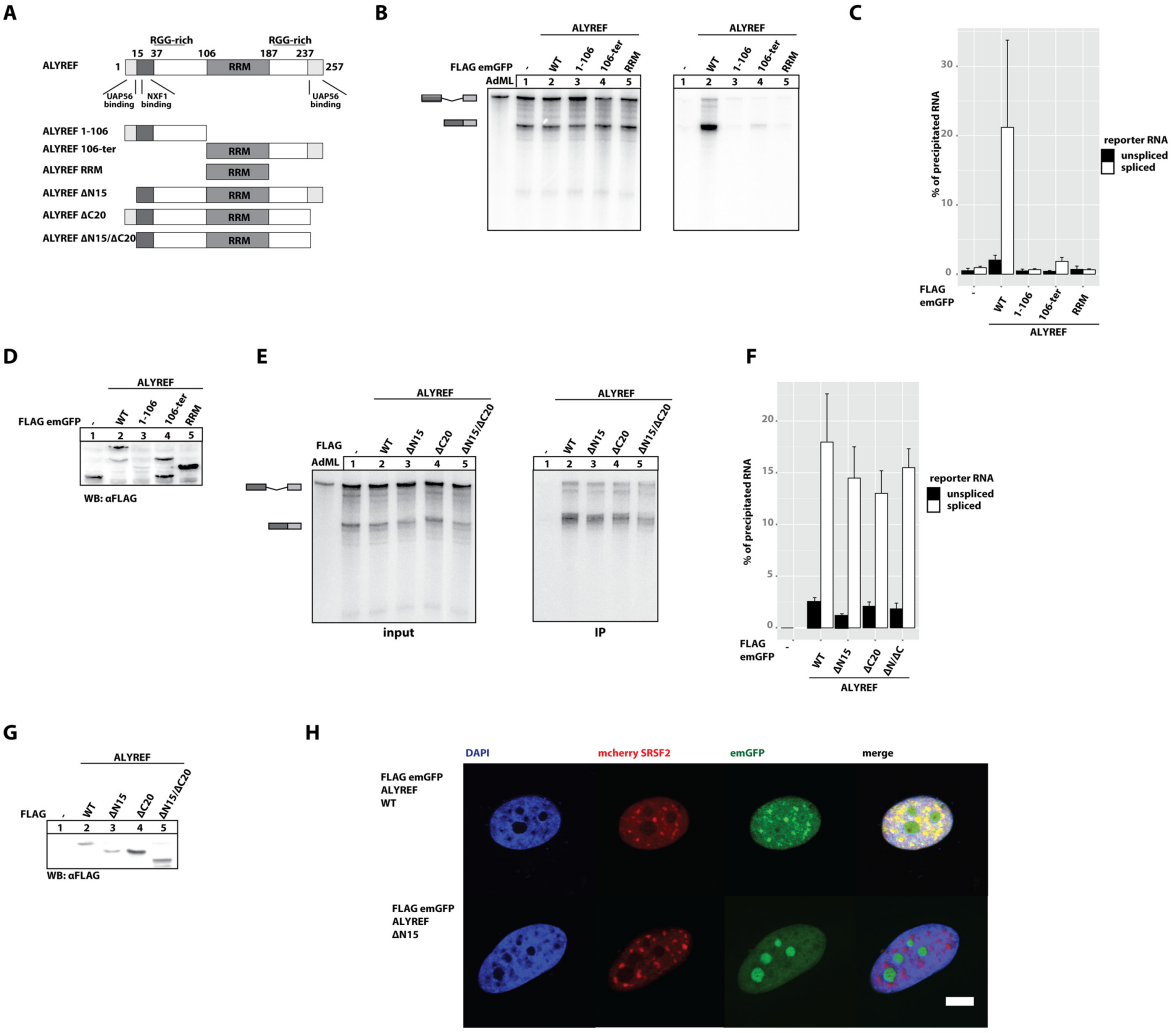
ALYREF binding to RNA *in vitro* is not mediated by its canonical RNA binding domain and is independent of UAP56 binding. (**A**) Schematic representation of ALYREF wild-type and mutants used in the study. (**B**) *In vitro* splicing reactions of AdML RNA were supplemented with extracts expressing FLAG-emGFP ALYREF truncation mutants (as in Figure 1A). Schematic representations of splicing and digestions products are depicted on the left side of the autoradiograph. (**C**) The results of three, independent, biological repetitions were quantified. Band intensities corresponding to the unspliced reporter RNA and spliced RNA are presented as black and white bars, respectively. (**D**) Expression of FLAG-tagged proteins in HEK 293 extracts used in (B) was determined by immunoblot analysis using a FLAG-antibody. (**E**) *In vitro* splicing reactions of AdML RNA were supplemented with extracts expressing FLAG-emGFP ALYREF truncation mutants as in (B). Schematic representations of splicing and digestions products are depicted on the left side of the autoradiograph. (**F**) The results of three independent biological repetitions were quantified and presented on the graph. Band intensities corresponding to the unspliced reporter RNA and spliced RNA are presented as black and white bars, respectively. (**G**) Expression of FLAG-tagged proteins in HEK 293 extracts used in (E) was determined by immunoblot analysis using a FLAG-antibody. (**H**) Localization of FLAG-emGFP ALYREF wild-type and mutant in HeLa cells. DAPI was used to stain nuclear DNA. Scale bar, 5 μm.

Inserts were transferred into various vectors in order to serve specific purposes. For protein expression in *in vitro* splicing experiments, constructs were inserted into the pCI-neo vector (Promega) with an N-terminal FLAG (DYKDDDDK) tag (Figures 1–3 and 5). ALYREF deletion mutants and the wild-type were cloned into pCI-FLAG-emGFP or into pCI-FLAG constructs for *in vitro* splicing (Figure 3). For localization studies, constructs were cloned into pCI-FLAG-emGFP (Figure 5E). For immunoprecipitation studies, constructs were cloned into pCDNA5 FRT/TO (Life Technologies) vector with an N-terminal triple FLAG-tag (Figure 5D). For export assays, pCI-Firefly and pCMV128 NanoLuc were used. pCMV128 NanoLuc was generated by inserting the NanoLuc sequence (Promega) into the NotI and BamHI sites of pCMV128 (32) (Figure 6).

Templates for *in vitro* transcription (MINX, AdML) of radiolabelled RNA were cloned into pGEM-4Z (Promega). MINX was described previously (31). AdML and AdMLΔi were generated by PCR amplification of a synthetic DNA (Figures 1–3 and 5). All constructs were verified by DNA sequencing.

### Bacterial expression

Recombinant PYM (rPYM) was obtained as described previously (31). Briefly, full-length PYM fused to a C-terminal Strep-tag was expressed in BL21 (DE3) bacteria and purified on StrepTactin columns. Purified PYM was desalted by ultrafiltration and diluted in buffer E.

### Cell culture and plasmid transfections

HEK 293 cell lysates expressing FLAG-tagged proteins for *in vitro* splicing assays were obtained as described previously (33). For imaging experiments, HeLa cells were transfected with 250 ng of FLAG-emGFP constructs and 250 ng of mCherry-SRSF2 in 6-well plates using 2X BES-buffered saline (BBS)-based calcium phosphate precipitation (34). For luciferase assays HeLa cells were transfected with 2 μg of FLAG-emGFP constructs, 500 ng of pCMV128 NanoLuc and 50 ng of pCI-Firefly constructs in 6-well plates using 2X BES-buffered saline (BBS)-based calcium phosphate precipitation (34).

The Flp-In T-Rex System (Life Technologies) was used to generate tetracycline-inducible, stable mammalian cell lines. Transgene expression was induced by adding 1 μg/ml doxycycline to the cell culture medium 72 h prior to harvesting. For immunoprecipitation experiments, HeLa cells were cultured and transfected as described previously (35).

### *In vitro* splicing assay

*In vitro* splicing assays were performed as described previously (33). Briefly, radiolabelled RNA substrates were incubated under splicing conditions in HeLa nuclear extract supplemented with whole-cell extracts from HEK293 cells expressing FLAG-tagged proteins. After splicing, RNA-protein complexes were immunoprecipitated via the FLAG-tagged proteins. The RNA was purified and subsequently analysed by denaturing gel electrophoresis and phosphorimaging.

For the cap competition assays shown in Figure 1C and D, splicing reactions were supplemented with 10 μM of m^7^GpppG RNA cap analogue. After splicing, FLAG-tagged proteins were immunoprecipitated and associated RNA was isolated as described above.

For RNAse H digestion of *in vitro* splicing assays (Figure 1E–G and Supplementary Figure S1) 1 μM of a cDNA oligonucleotide was added after the splicing reaction was completed. Digestion was performed for 15 min at 30°C. After splicing and digestion, RNA-protein complexes were immunoprecipitated via the FLAG-tagged protein. RNA was isolated and analysed as described above.

Oligonucleotides used for the oligo-directed digestion of spliced RNAs are listed below.

**Table:** 

Name	Sequence
MINX	5′ GAGTGGGCGAGC 3′
AdMl -36	5′ CGGAAGAGAGTG 3′
AdMl -58	5′ CGCGACGTCTCG 3′
AdMl -70	5′ ACCTGCAGGCCA 3′

In the splicing assay with rPYM, splicing reaction was performed as described above in addition of 1 and 2.5 μg of rPYM (Figure 6A, two concentrations). In Figure 6B, 2.5 μg of rPYM was used.

### Immunoblot Analysis and Immunoprecipitation

Immunoblot analysis and immunoprecipitation were performed as described previously (36). Briefly, Magnetic M2 anti-FLAG beads (Sigma-Aldrich) were used to immunoprecipitate FLAG-tagged complexes from RNase A-treated (50 μg/ml) HeLa cell lysates in lysis buffer (50 mM Tris [pH 7.2], 150 mM NaCl and 0.5% Triton X-100) supplemented with protease inhibitor (Sigma-Aldrich). Complexes were eluted with SDS-sample buffer, separated by SDS-PAGE and analysed by immunoblotting.

### Antibodies

FLAG-antibodies were from Sigma. The antibodies against UAP56 and ALYREF were generated by Thermo Scientific using peptides (UAP56: ENDVDNELLDYEDDEVET; ALYREF: CEELDAQLDAYNARMDTS) corresponding to amino acids 3–20 of human UAP56 and 241–257 of human ALYREF, respectively. The eIF4A3 polyclonal antibody was made by GenScript with an N-terminal peptide of eIF4A3 (37). The antibody against CBP80 was kindly provided by Elisa Izaurralde.

### Microscope image acquisition

Images were obtained at room temperature on an Olympus FV1000 confocal microscope using a 60x UPlanApo (NA 1.35) objective. Assembly, contrast and brightness adjustments of final image sets were performed using Photoshop CS4 (Adobe Systems, Inc.)

### Localization studies

Cells were seeded onto coverslips 24 h post-transfection, fixed with 3.7% formaldehyde (Carl Roth) in phoshate buffered saline (PBS) after 48 h and permeabilised for 5 min with 0.5% Triton X-100 (Sigma-Aldrich) in PBS on ice. Nuclei were counterstained with DAPI (Carl Roth). Coverslips were mounted in DABCO:Moviol (Carl Roth).

### Luciferase assay

Cells were harvested 72 h post-transfection using 300 μl Passive Lysis Buffer (Promega). Lysates (2 μl) were analyzed using the NanoGlo DLR Assay (Promega) on a Centro XS3 LB960 microplate luminometer (Berthold Technologies).

### Motif discovery

**Figure 4.**
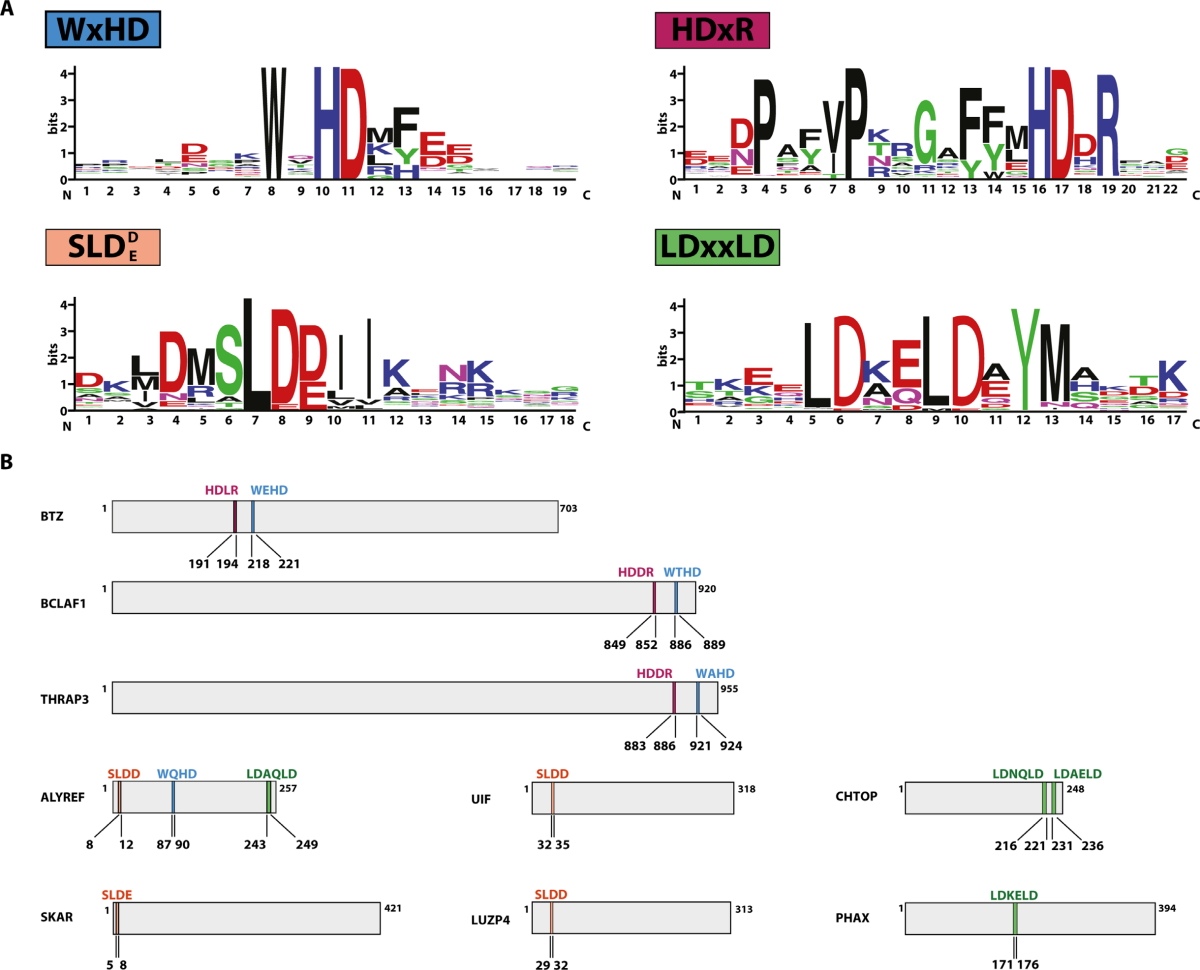
Short, conserved motifs present in RNA-binding proteins. (**A**) WebLogo diagrams of short conserved motifs identified in RNA-binding proteins. (**B**) Schematic representation of RNA-binding proteins and the distribution of short, conserved motifs listed in (A) (as above: WxHD motif in blue, HDxR motif in purple, SLDD/E motif in orange and LDxxLD motif in green). The sequence of the motif is indicated above the bar representing the protein, the position of the motif is indicated below the bar.

For computational motif discovery, a set of ALYREF orthologues from various metazoan species was subjected to multiple alignment by the L-INS-I method of the MAFFT software (38). The resulting alignment was used for the construction of a generalised profile (39) and iterative database searches in the UNIPROT database, always including hits with a *P*-value better than 0.01 for subsequent iteration cycles. An analogous procedure was used for finding sequence conservation in BTZ, BCLAF1, THRAP3, UIF, CHTOP, SKAR, LUZP4 and PHAX. When it became clear (usually after 1–3 iteration cycles) that the sequence conservation was restricted to one or more short motif-size regions, the multiple alignments where cropped and (if necessary) split, continuing the iteration cycle with one multiple alignment per conserved motif. Iterative refinement was stopped when no new significant hits were found in the database. The sequence logo representation shown in Figure 4 was generated using the WebLogo software (40). Finally, the relationship of the motifs to each other was assessed by using the HHSEARCH software (41).

## RESULTS

### Export complex components specifically bind to mRNA containing both cap and EJC

The recruitment of mRNA export factors to any given mRNA is a prerequisite for mRNA export and likely influences the efficiency and pathway of mRNA transport. Therefore, we aimed to understand how a subset of mRNA export factors interacts with mRNAs. We utilised a well-established *in vitro* splicing system, which has been shown to recapitulate the binding of different mRNP components to intron-containing (spliced) and intronless (non-spliceable) AdML and MINX RNA model substrates (33,35). Briefly, HeLa nuclear extract was supplemented with splicing-competent whole-cell extracts from HEK293 cells expressing FLAG-tagged export components (DDX39, UAP56 and ALYREF), FLAG-tagged SELOR (the eIF4A3-interacting domain of the EJC protein Barentsz) and FLAG-tagged CBP80. Subsequent to the splicing reaction, the FLAG-tagged proteins were immunoprecipitated together with their associated mRNAs (Figure 1A).

In this assay, ALYREF precipitated comparable amounts of spliced RNA as SELOR (the eIF4A3-interacting domain of Barentsz), indicating that it binds strongly to the spliced mRNA (Figure 1A, lanes 4 and 5). Barentsz (also referred to as BTZ, CASC3 or MLN51) is a component of EJC, known to bind spliced transcripts and therefore its EJC-binding domain SELOR was used as a positive control (35,42). Similar to ALYREF, UAP56 and DDX39 also bound spliced RNAs, but precipitated RNA less efficiently (Figure 1A, lanes 2 and 3). The binding pattern of the tested mRNA export components was different from the cap-binding protein CBP80. CBP80 bound preferentially to the cap and precipitated equal amounts of spliced and unspliced mRNA from *in vitro* splicing reactions (Figure 1A, lane 6). We also examined binding of export components to an intronless mRNA (AdML Δi), which was identical in sequence to spliced AdML RNA, but did not undergo splicing (Figure 1B). As expected, SELOR did not precipitate the intronless RNA (Figure 1B, lane 5). In contrast small amounts of AdMLΔi were precipitated by ALREF, UAP56 and DDX39, demonstrating their residual interaction with intronless RNAs. Nonetheless, these export factors appear to preferentially bind to the spliced mRNA, as reported previously (15–17). Notably, similar results were obtained with a MINX reporter RNA (data not shown).

Binding of export factors to the cap or the cap region of mRNAs has been previously demonstrated (16), but contradicting results were reported regarding the cap-dependent interaction of UAP56 and ALYREF with spliced mRNA (16,17). To investigate the role of the cap, we analysed if the presence of cap analogue (m^7^GpppG) interferes with binding of UAP56 and ALYREF to spliced mRNA (Figure 1C and D, respectively). In contrast to previous reports, the addition of cap analogue to the *in vitro* splicing reaction did not decrease splicing efficiency (17,43–44). Strikingly, binding to spliced RNA of both ALYREF and UAP56 was strongly impaired in the presence of cap analogue (compare lane 2 and 3 in Figure 1C and D), demonstrating that this interaction requires cap-binding proteins. This result is in line with the previously reported recruitment of the mRNA export machinery to the 5′ end of mRNA (16).

To further examine the importance of 5′ cap in binding of export components to the spliced RNA and to determine the position, where the core export components bind onto the RNA, we used oligonucleotide-directed RNase H cleavage of the mRNA after *in vitro* splicing assay. A similar assay was previously used to analyse the binding of the TREX complex to spliced RNAs (16). In our experiment, spliced and unspliced reporter mRNAs were cleaved at a position upstream of the EJC binding site in the first exon, which enabled us to separately study the binding of export factors to the cap or the EJC (Figure 1E, scheme). While the uncleaved spliced mRNA contains both the cap and EJC, the 5′ fragment of spliced mRNA contains only the cap, but no EJC, and the 3′ fragment of spliced mRNA contains the EJC, but not the cap (Figure 1E, scheme). Although DDX39, UAP56 and ALYREF efficiently bound to the full length spliced RNA, neither of the two fragments produced after RNAse H digestion was efficiently precipitated (Figure 1E, lanes 2–4). In contrast, SELOR precipitated the 3′ fragment with the EJC binding site (Figure 1E, lane 5), and CBP80 precipitated the cap-containing 5′ fragment (Figure 1E, lane 6), in line with their expected binding preferences. The expression of the FLAG-tagged proteins used in panels A–E was determined with Western blotting (Figure 1H).

To quantify the effect of cooperative binding to spliced mRNAs containing both the EJC and the cap, three independent biological repetitions were performed and quantified. In Figure 1F we calculated the ratio between the uncleaved spliced mRNA, containing both the cap and the EJC, and the cleaved mRNA containing only the cap (Figure 1F, Supplementary Figure S1A for alternative graphs). This ratio was set to 1 for CBP80, which does not bind to the cleaved mRNA containing only the EJC (Figure 1F), and therefore binds to mRNA based only on the presence of the cap. All tested export factors showed an enrichment in cooperative binding to uncleaved spliced mRNA compared to CBP80 (Figure 1F). Reciprocally, in Figure 1G we calculated the ratio between the uncleaved spliced mRNA and the cleaved mRNA containing only the EJC (Figure 1G, Supplementary Figure S1). This ratio was set to 1 for SELOR, which does not bind to the cleaved mRNA containing only the cap (Figure 1E), and therefore binds to mRNA based only on the presence of the EJC. All tested export components showed enrichment in cooperative binding to the uncleaved spliced mRNA compared to SELOR (Figure 1G). Notably, ALYREF showed the strongest enrichment in binding to spliced RNA for both parameters (>4-fold) (Figure 1F and G).

To validate that the observed differential precipitation of cleavage products is independent of the RNA sequence, we repeated the experiment using a different splicing substrate (AdML, Supplementary Figure S1B–D). As shown before for the MINX splicing substrate, DDX39, UAP56 and ALYREF only precipitated RNA fragments that contained both the cap and an EJC-binding site. Moreover, differential precipitation of cleavage products is also independent of the RNA length, as demonstrated by the use of DNA oligos that target different positions in the spliced RNA (Supplementary Figure S1B–D). Interestingly, binding of ALYREF was stronger on long cap-containing mRNA fragments. This is in agreement with previous studies suggesting that an RNA of sufficient length can enter the mRNA export pathway more readily than short and structured RNAs (45).

It has been reported that the presence of either the cap or the EJC increases binding of export factors to mRNA. However, it was not known if the simultaneous presence of both EJC and cap confers cooperative binding. Our results suggest that 5′ cap and EJC-binding site have an additive effect on the interaction of export factors with spliced mRNA.

### ALYREF binding to spliced mRNAs *in vitro* requires the EJC

To test if the association of ALYREF with spliced mRNA involves the EJC, we investigated the influence of PYM on binding of export components to mRNAs. PYM directly binds to the MAGOH-Y14 heterodimer to dissociate EJCs from mRNAs in the cytoplasm to promote recycling of EJCs (31,46). *In vitro*, the displacement of EJCs can be recapitulated by the addition of rPYM to splicing reactions (Figure 2B, scheme). We observed that UAP56 binding to spliced mRNAs was not affected by the addition of rPYM, whereas there was a slight decrease in RNA precipitation through DDX39 in the presence of rPYM (Figure 2A, lanes 1–7). In contrast, ALYREF binding to spliced mRNAs was significantly reduced after EJCs were disassembled (Figure 2A, lanes 8–10), albeit to a lesser extent than the binding of Y14, a core component of the EJC (Figure 2A, lanes 11–13). This observation suggests that ALYREF requires the interaction with an EJC to be recruited to spliced mRNAs.

To further validate that PYM-dependent reduction in RNA precipitation is mediated by the EJC, we performed a splicing assay followed by RNAse H digestion (as in Figure 1E) in the presence of rPYM (Figure 2B). After splicing in the presence of rPYM and RNase H-directed RNA digestion, three main species of RNA are present: uncleaved, spliced RNA containing cap and exon-junction; cleaved, spliced RNA with exon-junction and cleaved, spliced RNA with the cap. As shown before, addition of rPYM to the splicing reaction led to a significant reduction in the precipitation of RNA fragments containing an EJC-binding site (Figure 2B). This effect was much more pronounced for RNA fragments that lacked the 5′ cap (Figure 2B). Interestingly, in the presence of rPYM, UAP56, DDX39 and ALYREF all displayed an equally reduced ability to precipitated RNA fragments lacking the 5′ cap, demonstrating that the interaction of the three export receptors with uncapped RNA species is mediated by the EJC. Taken together, our data show that displacement of EJCs through PYM reduces the ability of UAP56, DDX39 and ALYREF to precipitate spliced RNA and therefore establishes a role for the EJC in export receptor recruitment. However, in the presence of a 5′ cap, individual export receptors display variable levels of EJC-dependence, with ALYREF being most and UAP56 least dependent on the EJC.

### ALYREF binding to UAP56 is dispensable for its interaction with mRNA *in vitro*

The recruitment of ALYREF to mRNAs by UAP56 has been reported to link pre-mRNA splicing and mRNA export (3,22). Therefore, we sought to study the function of the UAP56-binding regions of ALYREF in our splicing assay.

ALYREF contains an RNA-recognition motif (RRM; 106–187 aa), two previously described UAP56-interaction domains (UBM; N-terminal 1–15 aa, C-terminal 237–257 aa), an NXF1-binding motif (15–37 aa) and two arginine-rich regions (RGG; 17–87 aa; 198–231 aa) (3,13,22). Notably, the N- and C-terminal domains, but not the RRM, were sufficient for murine REF binding to RNA *in vitro* (47,48).

We constructed three large truncation mutants to investigate the mode of ALYREF interaction with mRNA. We deleted either the N-terminal part (106-ter aa), the C-terminal part (1–106 aa), or both parts of ALYREF (106–187 aa, corresponding to the RRM) (Figure 3A) and tested the RNA binding of these variants in our splicing assay (Figure 3B and C for quantification). The expression of ALYREF 106-ter and ALYREF RRM was comparable to the expression of full length ALYREF (Figure 3D). In contrast, we observed lower expression levels of the mutant ALYREF 1–106, which might be caused by the deletion of all known structured regions of ALYREF. None of the deletion mutants precipitated spliced RNA, suggesting that neither the RGG-rich regions nor the RRM is able to bind RNA by itself (Figure 3B). The substantial deletion of the C-terminal region of ALYREF (ALYREF 1–187) resulted in a milder reduction of binding to spliced and unspliced RNA (Supplementary Figure S2A), suggesting the RRM might mediate a weak interaction of ALYREF with both RNA species.

To correlate UAP56 interaction and RNA binding of ALYREF, we used mutants of ALYREF previously described to abolish binding to UAP56 (22) in order to investigate their interaction with RNA in the splicing assay (Figure 3E and F for quantification). We observed only a mild decrease of RNA binding with ALYREF lacking either the N-terminal (Figure 3E, lane 3) or the C-terminal sequence (Figure 3E, lane 4) or both (Figure 3E), even though these mutants display a strongly decreased ability to interact with UAP56 (Supplementary Figure S2B).

In conclusion, we show that neither of the potential RNA-binding domains of ALYREF (RGG, RRM) can mediate RNA binding in isolation. Furthermore, the deletion of the UAP56 binding sites maringally reduced the capability of ALYREF to bind spliced RNA *in vitro*, opposing the prevailing model that UAP56 is required for the deposition of ALYREF on spliced mRNAs.

In living cells, the recruitment of ALYREF to mRNAs during splicing is presumably executed in nuclear speckles (4,48–49). We performed co-localization studies using FLAG-emGFP ALYREF as well as the UAP56-binding deficient mutant deltaN15, together with the splicing factor (SRSF2, mCherry fusion), a marker for nuclear speckles (Figure 3H), and confirmed their co-localization (upper panel). Strikingly, the ALYREF mutant lacking the N-terminal UAP56 binding domain does not show speckle localization, but instead forming aggregates in the nucleoli, suggesting that while UAP56 is not required for RNA-loading of ALYREF, it plays a role in the proper localization of ALYREF to nuclear speckles.

### Proteins involved in mRNA export and mRNA processing contain various, short, conserved motifs

Recently it was reported that many mRNA-interacting proteins are intrinsically disordered and enriched in repetitive amino acid motifs (28,50–51). ALYREF contains two presumably unstructured regions in the N- and C-terminus, which might harbour functionally important sequences. In order to identify motifs potentially involved in mRNA export, we performed a computational analysis using mRNP-related proteins subjected to multiple alignment by the L-INS-I method (see Materials and Methods). Indeed, we identified four conserved motifs with the core sequences SLDD/E, LDxxLD, HDxR and WxHD (where x represents a variable amino acid) (Figure 4A). In addition to the short conserved core sequences, some highly conserved amino acids are found at other positions within the identified sequences (e.g. for HDxR: P is always present at the position -8 and -12).

All proteins containing the SLDD/E motif have been previously described to be involved in mRNA export, i.e. ALYREF, SKAR, UIF and LUZP4 (Figure 4B). The motif has been previously designated UBM (UAP56 binding motif), since it mediates the interaction with UAP56 (22,24,52).

The LDxxLD motif is present in ALYREF, PHAX and twice in CHTOP. In ALYREF this motif is part of the C-terminal UAP56-binding motif (22). CHTOP was found to be involved in mRNA export through the direct interaction with UAP56 (23). PHAX is involved in the export of snRNA and snoRNA through binding to the cap binding complex (CBC) (53,54). In contrast to mRNA export, which requires NXF1/NXT1, snRNA and snoRNA export involves CRM1 (a nuclear export receptor of the importin β family) and RanGTP. It is interesting to note that two conserved motifs required for UAP56-binding are found in proteins, which are implicated in the export of different classes of mRNAs. However, only ALYREF contains both motifs, whereas all other proteins have a single motif.

The SLDD/E and the LDxxLD motifs described above were previously reported to be variants of the same motif (22). Although we identified these sequences independently from each other, both motifs allow proteins to bind UAP56 and therefore execute the same molecular function.

Another short conserved motif present in ALYREF (WxHD) was found in BTZ, a component of the EJC core (8,42), and the two splicing factors BCLAF1 and THRAP3 (55,56). In the case of BTZ, BCLAF1 and THRAP3, the motif is preceded by another short motif HDxR. The function of these motifs remains unknown. However, the sequence WxHD of BTZ was previously identified to mediate binding to eIF4A3. Mutations within this motif (W218D; HD220AK) abolish the interaction with eIF4A3 and prevent BTZ from binding to mRNA and to other EJC components (35).

### ALYREF contains a short motif, conserved in other RNA-binding proteins, required for interaction with mRNA *in vitro*

Since ALYREF binding to mRNA does not involve the RRM and is independent of the presence of UAP56-binding motifs, we hypothesised that the conserved WxHD motif could contribute to mRNA binding of ALYREF (Figure 4).

**Figure 5.**
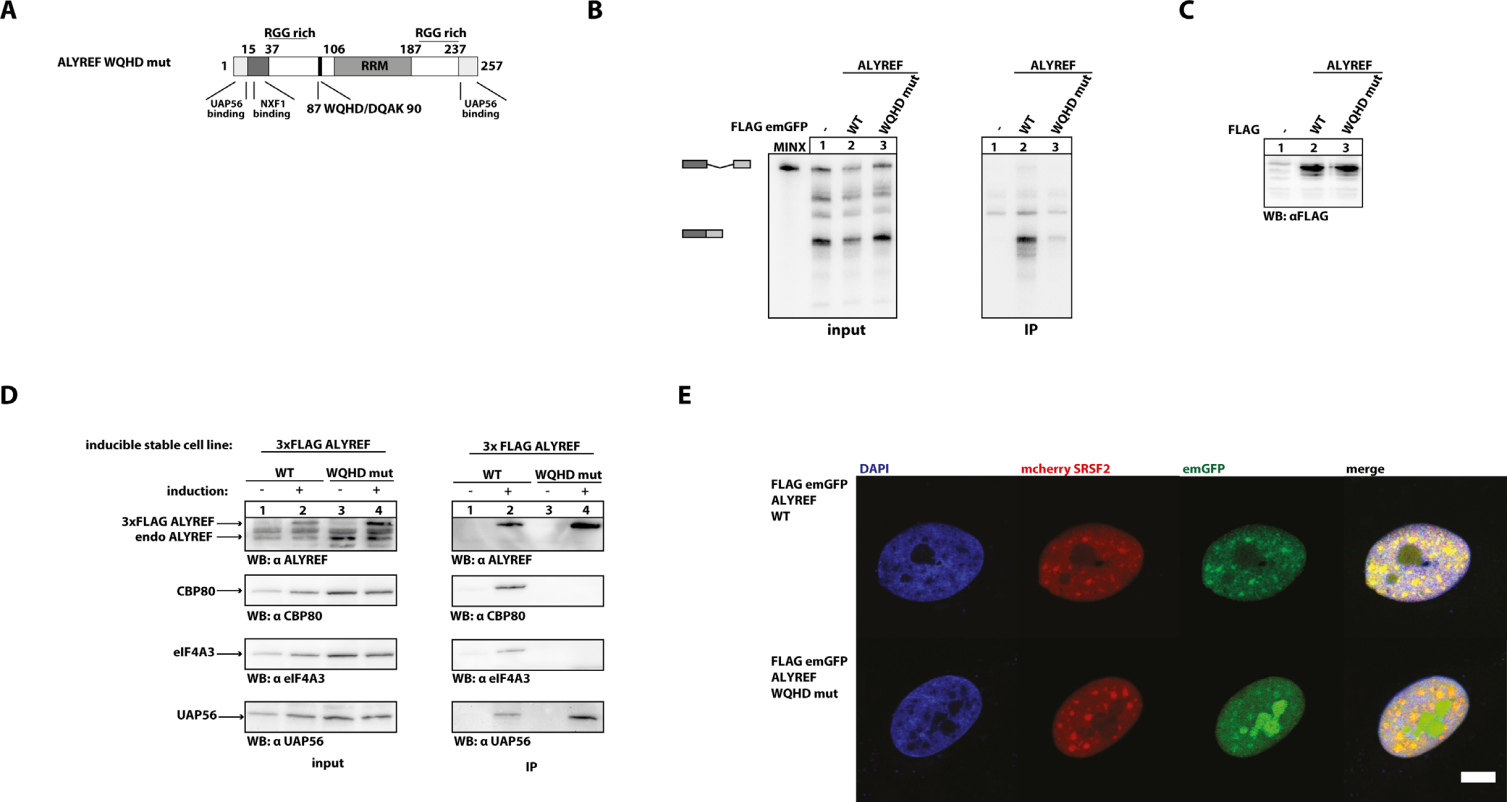
The WQHD motif in ALYREF is important for its interactions with spliced mRNA, eIF4A3 and CBP80. (**A**) The WQHD motif in ALYREF is placed in a low-complexity region. In the ALYREF point mutant used in this study, the WxHD motif is replaced by DQAK. (**B**) *In vitro* splicing reactions of MINX substrate were supplemented with extracts expressing FLAG-tagged ALYREF wild-type and DQAK mutant (as in Figure 1A). Splicing products are schematically represented on the left side of a gel. (**C**) Expression of FLAG-tagged proteins in HEK 293 extracts used in (B) was determined by immunoblot analysis using a FLAG-antibody. (**D**) FLAG-immunoprecipitations of RNase A-treated lysates from stable cell lines with induced expression of 3x FLAG ALYREF WT and WQHD mutant. Co-immunoprecipitated CBP80, eIF4A3 and UAP56 were detected by immunoblotting using a CBP80-, eIF4A3- and UAP56-specific antibodies, respectively. A total of 5% of cell extracts were loaded as input. Extracts without induced expression of 3xFLAG-tagged proteins were used as controls. (**E**) Localization of FLAG-emGFP ALYREF mutants in HeLa cells. DAPI was used to stain nuclear DNA. Scale bar, 5 μm.

Strikingly, a mutant of ALYREF, in which WQHD has been replaced by DQAK (Figure 5A) showed drastically reduced ability to co-immunoprecipitate mRNA generated by *in vitro* splicing (Figure 5B, compare lane 2 and 3), despite being expressed at similar levels (Figure 5C).

Since ALYREF preferentially interacted with spliced and capped RNAs *in vitro*, we wanted to study whether the WxHD motif is involved in the interaction with proteins of spliced mRNPs. To this end, cell lines expressing either 3xFLAG ALYREF WT or WQHD mutant were subjected to FLAG co-immunoprecipitation experiments. In RNase-treated lysates 3xFLAG ALYREF WT precipitated endogenous eIF4A3, CBP80 and UAP56. However, the 3x FLAG ALYREF WQHD mutant precipitated only UAP56, whereas the interaction with CBP80 and eIF4A3 was lost (Figure 5D).

To further test whether mutation of the WxHD motif affects the subcellular localization of ALYREF, we studied its colocalization with the splicing factor SRSF2 using fluorescently labelled proteins (Figure 5E). Interestingly, we observed a partial mislocalisation of the WQHD mutant. Although partial co-localization with SRSF2 was detectable, a substantial amount of ALYREF formed aggregates that did not colocalise with SRSF2, suggesting that RNA binding of ALYREF plays an important role in the proper targeting of the protein to nuclear speckles.

### The WQHD motif in ALYREF is required for efficient expression of spliced mRNAs *in vivo*

**Figure 6.**
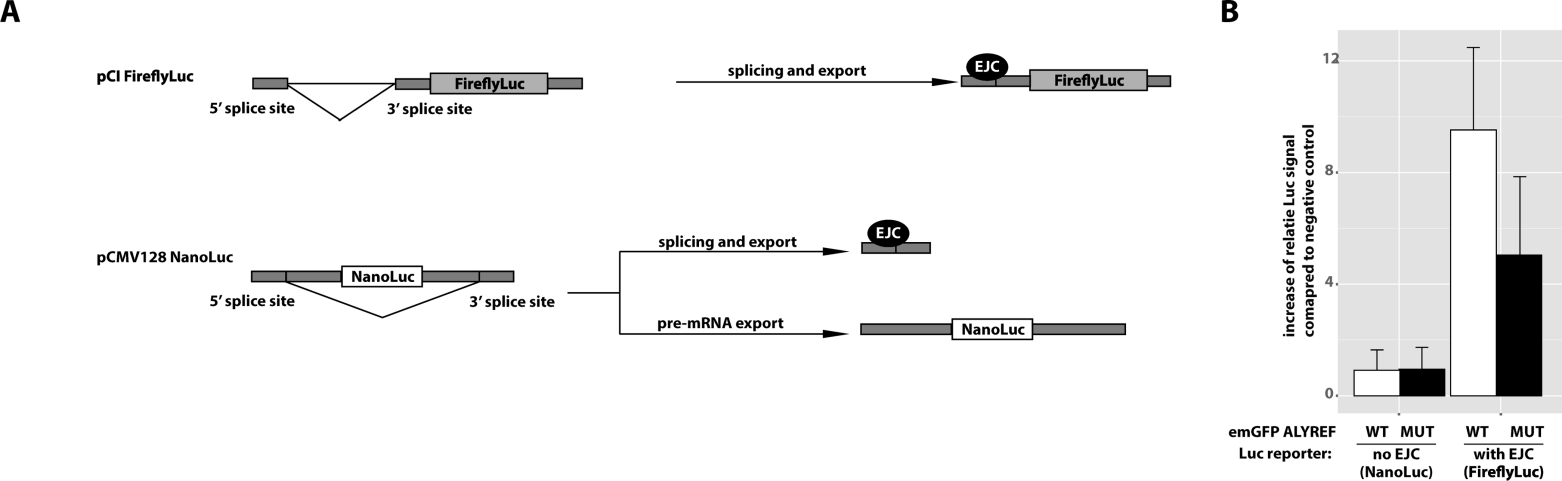
The WQHD motif in ALYREF is required for efficient export of spliced mRNAs *in vivo*. (**A**) Schematic representation of luciferase reporters used in the export assay (B). (**B**) Quantifications of three independent biological repetitions of luciferase export assay.

In order to test the biological significance of the identified WQHD motif in ALYREF, we studied the ability of ALYREF to promote the expression of a reporter mRNA in cultured cells (Figure 6). Briefly, HeLa cells were transfected with FLAG-emGFP ALYREF wild-type, WQHD mutant or FLAG-emGFP as negative control. Additionally, cells were cotransfected with two reporter constructs: pCI-FireflyLuc and pCMV128-NanoLuc. Importantly, FireflyLuc pre-mRNA is spliced in the nucleus (EJC is recruited), exported and translated (Figure 6A). In contrast, the open reading frame of the NanoLuciferase in pCMV128 NanoLuc is placed between two weak splice sites and can only be translated when the unspliced pre-mRNA is transported to the cytoplasm (57). If the pre-mRNA is spliced, the NanoLuc coding sequence is removed and no luciferase can be expressed (57).

Consistent with previous results showing that the presence of EJC promotes export and translation in an ALYREF-dependent manner, overexpression of ALYREF led to an increase in Firefly luciferase (spliced transcript, with EJC) compared to NanoLuc (unspliced transcript, no EJC). This effect was much less pronounced upon overexpression of the WQDH mutant of ALYREF, indicating that the WQHD motif plays an important role in the export of spliced mRNAs to the cytoplasm.

## DISCUSSION

In this study, we have re-examined the role of the cap and the EJC for export factor recruitment in human cells. We show that an excess of free cap analogue (m^7^GpppG) dissociates UAP56 and ALYREF from spliced RNA (Figure 1C and D), in agreement with the known requirement of a 5′ cap for their recruitment (17). We then exploited partial RNAse H digestion to examine if simultaneous presence of both the 5′ cap and the EJC is required for binding of export factors. Neither of the cleaved products containing either the cap or the EJC alone was precipitated to a similar extent as the uncleaved RNA, which contains both components (Figure 1E–G). We therefore conclude that recognition of spliced mRNAs by the core export factors requires cooperative binding to the cap and the EJC.

We also show that ALYREF binding to the spliced RNA is dependent on the presence of the EJC (Figure 2A and B). The dissociation of EJCs from RNA resulted in a significant decrease of ALYREF binding to spliced RNA, suggesting a functional connection between EJC and ALYREF. Furthermore, the EJC also contributes to binding of UAP56 and DDX39 to spliced mRNA. This contribution becomes only evident, when mRNA is cleaved into cap- and EJC-containing fragments (Figure 2B). These data suggest that UAP56 and DDX39 are more cap- than EJC-dependent, whereas ALYREF shows a more cooperative interaction with spliced mRNA.

### ALYREF binding to spliced mRNA is independent of UAP56

ALYREF contains different putative RNA-binding regions, of which RRM and RGG-rich regions of ALYREF were previously suggested as RNA-binding domains in living cells (58). However, we did not detect any interaction of the isolated RRM or RGG-rich regions with RNA. We also investigated the importance of the interaction of ALYREF and UAP56, since it was previously reported that ALYREF is loaded by UAP56 onto mRNA (3–4,15,18). Both, N-terminal and C-terminal UBM of ALYREF are involved in UAP56 binding (21). In contrast to previous findings, mutants of ALYREF unable to bind to UAP56 still retained the ability to associate with spliced RNAs *in vitro*, indicating that the interaction of ALYREF with mRNA occurs in a UAP56-independent manner. However, we report that the N-terminal UAP56-interaction motif is required for localization to nuclear speckles (Figure 5E), highlighting the functional connection of spliceosomal components (e.g. UAP56) with export factors (e.g. ALYREF). Therefore, we hypothesise that recruitment of ALYREF to the spliceosome is an important function of UAP56 binding to ALYREF.

### A novel mRNA binding motif WQHD is required for ALYREF deposition on spliced mRNA *in vitro*

Although several different models have been proposed in the literature, the exact mechanism of ALYREF deposition on spliced mRNA remains unknown. Here, we report the identification of a short motif (WxHD) in ALYREF, which is also present in four other proteins involved in mRNP formation. In ALYREF, the WQHD motif is located at position 87–91 in an unstructured region between the N-terminal RGG-like domain and the central RRM. Mutation of this motif leads to reduced binding of ALYREF to spliced RNA and impairs the interaction with CBP80 and eIF4A3. In contrast the interaction with UAP56 is not affected (Figure 5). Hence, WxHD may represent a core sequence, which can promote binding to other mRNP components. Notably, the truncated ALYREF 1–106 protein contains the WxHD regions, but fails to interact with RNA. This indicates that the motif is necessary, but not sufficient for binding to our reporter RNA. The specificity of the interaction may be determined by the surrounding sequences and allow proteins with this motif to bind to a number of similar interaction partners.

The involvement of the WxHD motif in ALYREF for mRNA expression and likely mRNA export was shown using a luciferase-based mRNA export assay in living cells (Figure 6). Overexpression of wild-type ALYREF promoted the expression of spliced reporter RNA, suggesting that the WQHD motif is required for the interaction between the export adaptor ALYREF and the EJC, and thereby for efficient export of spliced transcripts. Unspliced RNAs lack an EJC, they are not expected to bind ALYREF, which explains why the mutation in ALYREF has no effect on the export of the unspliced transcript (pCMV128 NanoLuc). This does not contradict the results of tethering experiments, which show that ALYREF can increase the export of unspliced reporter RNA when it is artificially tethered with boxB sequences (57). We confirmed that in the absence of tethering, the mutation of the WQHD motif disrupts the interaction of ALYREF with the EJC, and therefore only affects export of spliced, but not unspliced transcripts (Figure 6B).

### Novel short linear motifs are present in proteins involved in export of RNAs

In total, we identified four novel motifs that are present in proteins involved in mRNA export (ALYREF, CHTOP, LUZP4, PHAX, SKAR and UIF), which might belong to the growing family of SLiMs, also referred to as ELMs (28,59). SLiMs are short (3–10 amino acids) and present in disordered regions (also referred to as low-complexity regions, LC), and are expected to adopt a defined conformation only upon binding to their interaction partner (28). In contrast to protein–protein interactions mediated through folded domains, SLiMs likely interact with relatively low affinity with their binding partner, leading to transient interaction networks that can be regulated by post-translational modifications. Higher specificity and affinity are achieved by combining motifs within one protein, by additional interactions involving the flanking regions or by oligomerisation of several binding partners. Functionally, SLiMs either bind surfaces of the folded domains of their binding partner or intrinsically form complex mRNP granules (28,60). Our bioinformatic analysis suggests that various export adaptors bind to common interaction partners during the export of specific classes of mRNAs (22,24). The combination of different motifs may determine the specificity of a given export adaptor for certain mRNA targets, but may also be responsible for the observed plasticity of the network of mRNA export factors.

Notably, the function of two motifs (HDxR and WxHD) in two additional proteins – BCLAF1 (Bcl-2-associated transcription factor 1, also referred to as BTF) and THRAP3 (Thyroid hormone associated receptor 3, also referred to as TRAP150) has not been determined yet. BCLAF1 and THRAP3 were initially described as spliceosomal components (61), both were detected in the EJC-interactome and colocalise with EJC components in the cell (62,63). Moreover, RNA-like aggregates precipitated by biotinylated isoxazole contained proteins with WxHD motifs (e.g. ALYREF, THRAP3 and BTZ) (51,64). Notably, THRAP3 was identified as an NXF1 interaction partner and was reported to be involved in nuclear degradation of mRNA, suggesting a link between THRAP3 and RNA export machinery (55).

### Implications for mRNA export

It has been speculated that splicing and deposition of EJCs enhance recruitment of export components and thereby stimulate export of spliced mRNPs (7). The EJC protein BTZ is localised to the cytoplasm in steady state and therefore may only join the EJC after export. Hence, the surface patch of eIF4A3 that is used for interaction with BTZ is not occupied in the nucleus. Therefore, it is feasible that ALYREF docks onto eIF4A3 as part of the EJC in the nucleus after splicing is completed. However, a stable interaction of ALYREF with the mRNA requires the cap, which provides an additional binding site. This is in line with the previous observation that export complexes are only located between cap and first EJC in mRNA containing many EJCs (16). Finally, the export-competent mRNP is transported to the nuclear pore by a transient interaction with NXF1. ALYREF and other export factors are disassembled from the mRNP and the eIF4A3 binding site can interact with BTZ in the cytoplasm. Alternatively, an EJC-independent recruitment of ALYREF to mature mRNAs may involve direct binding of the WxHD motif to RNA. In either case, it will be interesting to find out how the simultaneous interaction of ALYREF with two distinct regions of the mRNA is established.

*In vivo*, the assembly of export complexes on mature mRNA may involve additional components of the mRNP, which have not been tested in this study. It has been shown, for example, that ALYREF interacts with the 3′ end processing factor Pcf11 (65), and UAP56 was co-purified with 3′ end processing complexes (66).

### Implications for neurodegenerative disease

Several components of export machinery have been implicated to play a role in the mechanism of amyotrophic lateral sclerosis pathogenesis (ALS)(67). ALS leads to the progressive loss of motor neurons and neuromuscular junctions. Around 10% of the familial form of ALS is caused by the hexanucleotide expansion (GGGGCC) in the C9ORF72 gene, leading to faulty mRNAs and toxic dipeptides (68,69).

The recently published screening for hexanucleotide repeats expansions toxicity modifiers identified the components of export machinery as potential candidates. In fruit fly, the deficiency of ALYREF supressed the toxicity of G4C2 expansions. This phenomenon might be explained by our observation that EJC-containing mRNAs are exported more efficiently when ALYREF is overexpressed (Figure 6). In contrast, mRNAs without EJCs (such as G4C2 repeats) are retained in the nucleus or not efficiently expressed (68,69). We postulate that ALYREF acts as part of a control mechanism that retains unspliced or faulty RNAs in the nucleus. Furthermore, ALYREF and its interaction with EJC through the low complexity region might also play a role as a regulator for RNA toxicity of C9ORF72 gene leading to neurodegeneration.

## Supplementary Material

SUPPLEMENTARY DATA
