# Supplementary material for: A short conserved motif in ALYREF directs cap- and EJC-dependent assembly of export complexes on spliced mRNAs
Source: Nucleic Acids Res. 2016 Jan 14;44(5):2348–61. doi: 10.1093/nar/gkw009 (PMC4797287; doi:10.1093/nar/gkw009)
Supplement: SUPPLEMENTARY DATA [file supp_44_5_2348__index.html]

A short conserved motif in ALYREF directs cap- and EJC-dependent assembly of export complexes on spliced mRNAs — A short conserved motif in ALYREF directs cap- and EJC-dependent assembly of export complexes on spliced mRNAs — SUPPLEMENTARY DATA 

# A short conserved motif in ALYREF directs cap- and EJC-dependent assembly of export complexes on spliced mRNAs

## SUPPLEMENTARY DATA

- SUPPLEMENTARY DATA
