# Supplementary material for: A short conserved motif in ALYREF directs cap- and EJC-dependent assembly of export complexes on spliced mRNAs
Source: Nucleic Acids Res. 2016 Jan 14;44(5):2348–61. doi: 10.1093/nar/gkw009 (PMC4797287; doi:10.1093/nar/gkw009)
Supplement: SUPPLEMENTARY DATA [file supp_gkw009_nar-01255-a-2015-File008.pdf]

Supplementary Material

A short conserved motif in ALYREF directs cap- and EJC-dependent assembly of export complexes on spliced mRNAs

Agnieszka M. Gromadzka , Anna-Lena Steckelberg, Kusum K. Singh, Kay Hofmann, Niels H. Gehring

Supplementary Figure 1

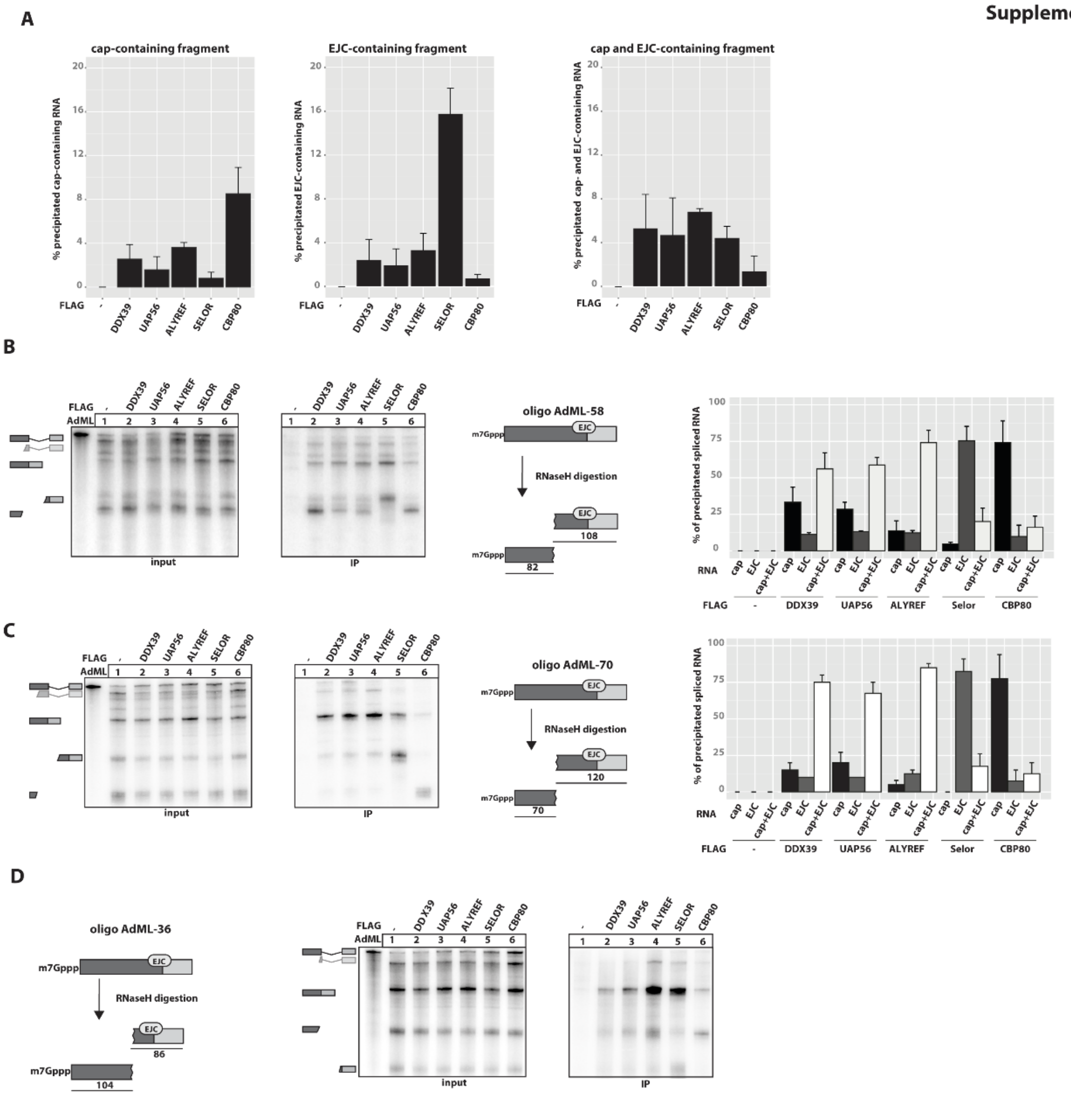

Supplementary Figure 1

A. Results of three independent biological repetitions of the experiment shown in Figure 1 C were quantified. The intensities of the cap-containing, EJC-containing and non-cleaved spliced bands are shown.

B. In vitro splicing reactions were performed as in Figure 1E using the AdML substrate. The oligonucleotide directing RNase H digestion was designed to cleave at a position 58 nucleotides downstream from the exon-junction. The splicing and digestion scheme is represented on the left side of the autoradiograph. Three independent repetitions of the experiment presented in this panel were quantified and presented in the graph.

C. In vitro splicing reactions were performed as in (A). The oligonucleotide directing RNase H digestion was designed to cleave at a position 70 nucleotides downstream from the exon-junction. Three independent repetitions of the experiment presented in this panel were quantified and presented in the graph.

D. In vitro splicing reactions were performed as in (A). The oligonucleotide directing RNase H digestion was designed to cleave at a position 36 nucleotides downstream from the exon-junction.

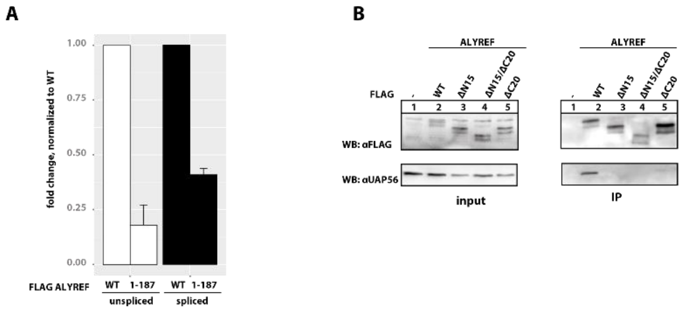

Supplementary Figure 2

Supplementary Figure 2

A. In vitro splicing reactions of AdML substrate were supplemented with extracts expressing FLAG-tagged ALYREF wild-type and 1-187 mutant (as in Figure 1). Three independent repetitions of the experiment were quantified and presented on the graph.

B. FLAG-immunoprecipitations of ALYREF mutants in RNase A-treated HeLa cell extracts. Co-immunoprecipitated UAP56 was detected by immunoblotting using a UAP56-specific antibody. 5% of cell extracts were loaded as input. Extract expressing unfused FLAG-tag served as a negative control.

Supplementary Figure 3

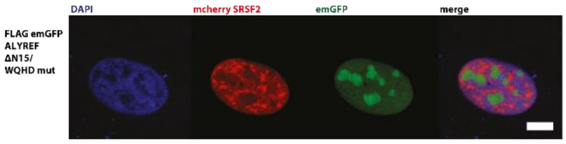

Supplementary Figure 3

A. Localization of FLAG-emGFP ALYREF mutant in HeLa cells. DAPI was used to stain nuclear DNA. Scale bar, 5 µm.
